# Non-targeted Metabolomics in Diverse Sorghum Breeding Lines Indicates Primary and Secondary Metabolite Profiles Are Associated with Plant Biomass Accumulation and Photosynthesis

**DOI:** 10.3389/fpls.2016.00953

**Published:** 2016-07-11

**Authors:** Marie F. Turner, Adam L. Heuberger, Jay S. Kirkwood, Carl C. Collins, Edward J. Wolfrum, Corey D. Broeckling, Jessica E. Prenni, Courtney E. Jahn

**Affiliations:** ^1^Department of Bioagricultural Sciences and Pest Management, Colorado State UniversityFort Collins, CO, USA; ^2^Department of Horticulture and Landscape Architecture, Colorado State UniversityFort Collins, CO, USA; ^3^Proteomics and Metabolomics Facility, Colorado State UniversityFort Collins, CO, USA; ^4^National Renewable Energy Laboratory, National Bioenergy CenterGolden, CO, USA

**Keywords:** *Sorghum bicolor*, GC-MS, LC-MS, biomass, metabolomics, photosynthesis, chlorogenic acid, shikimic acid

## Abstract

Metabolomics is an emerging method to improve our understanding of how genetic diversity affects phenotypic variation in plants. Recent studies have demonstrated that genotype has a major influence on biochemical variation in several types of plant tissues, however, the association between metabolic variation and variation in morphological and physiological traits is largely unknown. *Sorghum bicolor* (L.) is an important food and fuel crop with extensive genetic and phenotypic variation. Sorghum lines have been bred for differing phenotypes beneficial for production of grain (food), stem sugar (food, fuel), and cellulosic biomass (forage, fuel), and these varying phenotypes are the end products of innate metabolic programming which determines how carbon is allocated during plant growth and development. Further, sorghum has been adapted among highly diverse environments. Because of this geographic and phenotypic variation, the sorghum metabolome is expected to be highly divergent; however, metabolite variation in sorghum has not been characterized. Here, we utilize a phenotypically diverse panel of sorghum breeding lines to identify associations between leaf metabolites and morpho-physiological traits. The panel (11 lines) exhibited significant variation for 21 morpho-physiological traits, as well as broader trends in variation by sorghum type (grain vs. biomass types). Variation was also observed for cell wall constituents (glucan, xylan, lignin, ash). Non-targeted metabolomics analysis of leaf tissue showed that 956 of 1181 metabolites varied among the lines (81%, ANOVA, FDR adjusted *p* < 0.05). Both univariate and multivariate analyses determined relationships between metabolites and morpho-physiological traits, and 384 metabolites correlated with at least one trait (32%, *p* < 0.05), including many secondary metabolites such as glycosylated flavonoids and chlorogenic acids. The use of metabolomics to explain relationships between two or more morpho-physiological traits was explored and showed chlorogenic and shikimic acid to be associated with photosynthesis, early plant growth and final biomass measures in sorghum. Taken together, this study demonstrates the integration of metabolomics with morpho-physiological datasets to elucidate links between plant metabolism, growth, and architecture.

## Introduction

The increasing availability of tools such as transcriptomics, metabolomics, and proteomics has helped to facilitate the discovery of previously cryptic connections between genotype and phenotype. Metabolomics, in particular, provides an opportunity to examine a more nuanced phenotypic analysis in that it, while still removed from the visible phenotype, offers a chance at a more functional or mechanistic interpretation of species variation or plant responses at the molecular level (Feussner and Polle, 2015). Additionally, because small molecules are regulated by upstream gene expression and transcript formation, their measurement presents a complementary and functional approach to conventional genomics and transcriptomics (Bino et al., 2004). In this way, metabolomics offers benefits to both basic integrative fields such as systems biology as well as new ways to identify targets for improvement in applied disciplines such as plant breeding (Fernie and Schauer, 2009).

In recent studies, metabolomics has been used in such applications as characterization of biochemical variation within species (e.g., Kusano et al., 2015), discovery of potential metabolic engineering targets (Tsogtbaatar et al., 2015) and examination of plant responses to the whole environment (Steinfath et al., 2010; Heuberger et al., 2014a), as well as responses to individual biotic (e.g., Scandiani et al., 2015) and abiotic (Ganie et al., 2015; Sanchez-Martin et al., 2015) stressors. In addition, genome wide association mapping (GWAS) is being used to explicitly link the chemical diversity of metabolomic profiles with specific locations in the genome to help dissect quantitative traits (Riedelsheimer et al., 2012). Further, metabolic subsets are being explored as potential front-end tools such as biomarkers or for model-based prediction of traits that would otherwise require the high time and resource investments for one or more seasons of field trials (Meyer et al., 2007; Steinfath et al., 2010; Heuberger et al., 2014b).

Drought is the most costly abiotic stress for agriculture worldwide. Globally, many regions are expected to experience even more frequent and severe droughts in the coming century due to increasing atmospheric temperatures (Dai, 2011), making research on drought resistant crops a crucial component to improve food security or reduce costly inputs to biofuel production. Plants with a C4 photosynthetic pathway are particularly valuable in crop production due to their physiological advantage under hot and dry conditions (Taylor et al., 2014).

Sorghum [*Sorghum bicolor* (L.) Moench] is an internationally important C4 crop which produces grain, sugar syrup, and cellulosic biomass and can therefore be diverted to multiple markets, including food for human and animal consumption, and feedstock for various methods of biofuel production. This market flexibility is due to extensive phenotypic variation for the ways in which sorghum accumulates and allocates biomass to its leaves, stems, and panicles. Sorghum is also increasingly used as a model for other C4 species due to its small genome, available sequence, and annotation resources (Mace et al., 2013; Mullet et al., 2014). In addition, even within relatively limited breeding populations, sorghum is genetically diverse (Evans et al., 2013), with variation for agronomically important traits such as resistance to drought and tolerance of poor soils (Mace et al., 2013). Further, sorghum lines vary for photoperiod sensitivity, a foundational trait that enables breeders to shift carbon pools away from grain and toward vegetative tissues in plants well-suited for forage, biofuel feedstocks, or sugar (Rooney et al., 2007). Varieties that remain vegetative for longer periods of time maintain higher growth rates and can therefore accumulate up to 100% more biomass than grain-types that are quick to reach reproductive maturity (Mullet et al., 2014). Several morphological factors contribute to end biomass yield in sorghum, including variation in not only growth rate, but also allocation to different plant organs (leaves, stems, panicles). We define this collection of associated phenotypes (e.g., growth rate, harvest indices, final yield) as the process of “biomass accumulation.” Despite this morphological variation, sorghum can be broadly classified into two “types” based on allocation of carbon pools to major distinct tissues: (1) “grain type”: small plants bred for dense panicles, or (2) “biomass type”: large plants bred for total biomass (used as forage, sugar, or biofuels).

Because of the significant phenotypic variation in sorghum, it is reasonable to expect that metabolic variation among sorghum lines should also be high; however, this variation has yet to be characterized. This study described herein had two major objectives: (1) To examine and characterize the metabolic variation in an important set of sorghum breeding lines via non-targeted GC- and LC-MS analyses and (2) To explore the association of these metabolite profiles with an array of measured phenotypes (morphological, physiological, and structural carbohydrate content) expected to be relevant to plant growth, biomass accumulation, and biomass quality. Indeed, we found that both individual metabolites and profiles varied widely across lines and many small molecules had strong associations with morphological and physiological phenotypes.

## Materials and methods

### Plant materials and growth conditions

Eleven diploid lines from both “grain” and “biomass” type sorghums were selected to represent available variation and genetic tools [e.g., *BTx623* which is sequenced (Paterson et al., 2009), or *RTx430* which can be transformed (Wu et al., 2014)], making them valuable in pursuit of improved crops. Major characteristics and select publications related to this panel are presented in Table 1.

**Table 1:** **Line, type, characteristics, and associated publications for lines in this study**.

**Line**	**Type/use**	**Resources and characteristics**	**Selected publications**
BTx623	Grain	Sequenced; pre-flowering drought tolerant	Hart et al., 2001; Brown et al., 2006; Murray et al., 2008b; Paterson et al., 2009
RTx430	Grain	Used for transformation; post-flowering drought susceptible	Miller, 1984; MacKinnon et al., 1987; Howe et al., 2006; Liu and Godwin, 2012; Wu et al., 2014
IS3620C	Grain	Converted inbred	Brown et al., 2006; Burow et al., 2011
BTx642	Grain	Sequenced; post-flowering drought tolerant	Subudhi et al., 2000; Evans et al., 2013
Tx7000	Grain	Elite line; pre-flowering drought tolerant, Sequenced	Subudhi et al., 2000; Kebede et al., 2001; Evans et al., 2013
SC56	Grain	Stay green; pre-flowering drought susceptible	Kebede et al., 2001
SC170	Grain	Grain mold research cultivar	Little and Magill, 2009
M35-1	Biomass/grain	RIL parent	Reddy et al., 2012
100M	Biomass	Photoperiod sensitive NIL(Maturity)	Sorrells and Myers, 1982; Childs et al., 1992; Murphy et al., 2011
Rio	Biomass/sweet	Juicy-stalked; parent line	Broadhead, 1972; Murray et al., 2008a,b; Felderhoff et al., 2012
N598	Biomass/forage	Low-lignin mutant NIL (BMR)	Pedersen et al., 2006

NIL, Near Isogenic Line; RIL, Recombinant Inbred Line; BMR, Brown Mid Rib mutant (low lignin).

Seeds were germinated (25⋅C, dark) in Petri dishes on filter paper with fungicide solution (Maxim XL, Syngenta) for 1 week prior to transplanting to pots (3.8 L) filled with Fafard 4P all-purpose, high-porosity potting mix. Plants were grown in a controlled greenhouse environment (mean 25/20⋅C day/night; 38/51% relative humidity day/night). Five replicates of each line were randomized to minimize effects of varying conditions across the bench. Supplemental lighting maintained a 16/8h light/dark photoperiod and mean daytime PAR was 323 ± 52 μmol m^−2^s^−1^. Plants were checked daily to maintain benign (unstressed) conditions and watered when the top 2 cm of medium became dry. Starter nutrients were present in the potting medium but to avoid limitation, plants were fertilized one additional time at 7 weeks post-germination with Osmocote time release (14-14-14) at a rate of 18 g per pot.

### Phenotyping of morphological, physiological, and structural carbohydrate traits

Plants were evaluated starting at 28 days post-germination and growth rates were calculated as the difference between plant heights in consecutive weeks. At reproductive maturity for each line, the following measurements were made: plant height was measured as distance from potting medium surface to tip of tallest leaf. Stem diameter was measured directly above the first node on the main tiller with a digital caliper (Neiko). Leaf length and width were measured on the five most recently fully expanded leaves, averaged, and used to estimate area. At harvest, biomass was partitioned into leaves, stems, and panicles before oven-drying at 93⋅C until samples reached constant weight. Harvest indices were calculated as proportions (dry biomass of particular tissue type: total dry biomass).

Chlorophyll extraction was performed on the youngest, fully expanded leaf of 11 week-old plants, which was cut into pieces and ground into a fine powder under liquid nitrogen. Chlorophyll was extracted from 300 mg ground tissue in 50 ml 80% acetone at 4⋅C, with shaking at 125 rpm for 30 min in the dark. Debris were removed with centrifugation at 1280 relative centrifugal force (rcf) for 15 min at 4⋅C. Absorbance of the chlorophyll solution was measured using a Synergy HT Multi-Detection Microplate Reader (BIO-TEK Instruments, Inc., Winooski, VT, U.S.A.) at 645 and 663 nm. Chlorophyll content was estimated using the formula of (Arnon, 1949).

Gas exchange measurements were made in the greenhouse on vegetative, 6-week-old plants, on the youngest, fully expanded leaf. Measurements were made in randomized order within a midday 4-h window on 2 consecutive days using the LI-6400XT portable photosynthesis system (LI-COR, Inc.) with the leaf chamber fluorometer attachment. The cuvette was placed at the midpoint of the leaf, avoiding the midrib. Measurements were taken under the following conditions: leaf temperature = 25⋅C, photosynthetically active radiation (PAR) = 1500 μmol m^−2^s^−1^, CO_2_ = 400 μmol mol^−1^, and ambient RH = (38–42%). CO_2_ fixation rate, stomatal conductance, and Ci:Ca (ratio of intercellular to ambient CO_2_) were recorded and intrinsic water use efficiency (WUE) was calculated as a ratio of CO_2_ fixation to stomatal conductance.

For NIRS analysis, the stem fraction (at harvest) of dry biomass was ground with a Model 4 Wiley Mill (Thomas Scientific) to pass through a 2 mm screen. Samples were and analyzed via NIRS and a multiple-feedstock multivariate calibration model (Wolfrum et al., 2013; Payne and Wolfrum, 2015) was used to generate predicted percentages and uncertainties for glucan, xylan, lignin, and ash. The calibration model was developed using primary compositional analysis data on a wide variety of biomass samples (including corn stovers, sorghum, miscanthus, cool-season grasses, and switchgrass) using standard wet chemical techniques (Sluiter et al., 2010; Templeton et al., 2010). All samples passed quality control, and uncertainties (UC) in predictions of compositional data were characterized using the empirical U-deviation method (Zhang and Garcia-Munoz, 2009), which calculates multivariate confidence intervals (CIs), similar in principal to 95% CIs for linear models. Larger UC values indicate higher uncertainties in predicted values. Mass of each constituent was calculated as the decimal percentage multiplied by final biomass.

### Metabolite extraction

Leaf samples were collected from 4-week-old plants. One leaf from each of *n* = 5 plant replicates per breeding line was used. Tissue was excised from leaves via a cork borer. The tissue was collected in the same spot on each leaf replicate, from the middle of the fully developed sixth leaf beside, but not including the midrib. For each sample, ~25 mg tissue (dry weight) was immediately placed in a 1.5 mL tube containing stainless steel grinding balls, frozen in liquid N_2_, and homogenized using a paint-shaker. Metabolites were extracted by adding 1 mL of methanol:water (70:30, v:v) and vortexing for 2 h at room temperature. Samples were centrifuged at high speed (13,500 rcf, 10 min, 4⋅C), and 800 μL of supernatant was transferred to new tubes and stored at −80⋅C.

### Metabolite detection using GC-MS

Five hundred microliter of the metabolite extract was dried using a speedvac. Samples were derivatized by re-suspending the extract in 50 μL of pyridine containing 15 mg/mL of methoxyamine hydrochloride, incubating at 60⋅C for 45 min, sonicating for 10 min, and incubating again at 60⋅C for an additional 45 min. Next, 50 μL of N-methyl-N-trimethylsilyltrifluoroacetamide with 1% trimethylchlorosilane (MSTFA+1% TMCS, Thermo Scientific) was added and samples were incubated at 60⋅C for 30 min, centrifuged at 3000 × g for 5 min at 4⋅C, cooled to room temperature, and 80 μL of supernatant was transferred to a 150 μL glass insert. Metabolites were detected with a Trace GC Ultra coupled to a Thermo DSQ II (Thermo Scientific), acquiring mass spectra of 50–650 m/z at 5 scans s^−1^ in electron impact mode after separation on a 30 m TG-5MS column (Thermo Scientific, 0.25 mm i.d., 0.25 μm film thickness). Both inlet and transfer lines were set at 280⋅C. Samples were injected in a 1:10 split ratio twice in discrete randomized blocks with a 1.2 ml min^−1^ flow rate, following a program of 80⋅C for 30 sec, a ramp of 15⋅C per min to 330⋅C, and holding at 330⋅C for 8 min.

### Metabolite detection using ultra performance LC-MS

One microliter of metabolite extract was injected into an Acquity UPLC system (Waters Corporation). Separation was conducted with an Acquity UPLC T3 column (1.8 μm, 1.0 × 100 mm; Waters Co.), using a gradient from solvent A (water, 0.1% formic acid) to solvent B (acetonitrile, 0.1% formic acid). Injections were made in 100% A, which was held for 1 min, a 12 min linear gradient to 95% B was then applied, and held at 95% B for 3 min, returned to starting conditions over 0.05 min, and allowed to re-equilibrate for 3.95 min. Flow rate was constant (200 μl min^−1^) for the entire run duration. The column was held at 50⋅C with samples held at 5⋅C. Column eluent was coupled directly to a Xevo G2 Q-Tof MS (Waters Co.) fitted with an electrospray source. Data was collected in positive ion mode, scanning from 50–1200 at 5 scans s^−1^, alternating between MS and MS^E^ mode. Collision energy was set to 6 V for MS mode, and ramped from 15–30 V for MS^E^ mode. Calibration was performed prior to analysis via infusion of sodium formate solution, with mass accuracy within 1 ppm. Capillary voltage was held at 2200 V, source temperature at 150⋅C, and desolvation temperature at 350⋅C at a nitrogen desolvation gas flow rate of 800 L h^−1^.

### Metabolomics data analysis

For each sample, a matrix of molecular features defined by retention time and mass (m/z) was generated using XCMS software (Smith et al., 2006) using settings as previously described (Broeckling et al., 2014), independently for GC- and LC-MS data sets. Samples were normalized to total ion current and relative quantity of each molecular feature was determined by mean area of the chromatographic peak among replicate injections (*n* = 2). For LC- and GC-MS, mass spectra, and metabolite quantities were generated using an algorithm that clusters masses into spectra (“spectral clusters” that represent “compounds”) based on co-variation and co-elution in the data set (Broeckling et al., 2014), and were annotated by searching against in-house and external metabolite databases including NIST v12 (http://www.nist.gov), Massbank (http://www.massbank.jp), Golm (gmd.mpimp-golm.mpg.de), and Metlin (metlin.scripps.edu). Annotations using high resolution mass spectrometry (LC-MS) involved the identification of precursor ions within ~5 ppm error of the expected positive ion adduct (e.g., H^+^ or Na^+^). Glycosides were identified by neutral loss of 162.05 m/z in LC-MS spectral clusters.

### Statistical analysis

Analysis of Variance (ANOVA) for morphological and physiological traits was performed in JMP Pro 10 (SAS Institute). Prior to analysis, data were Box-Cox transformed to improve normality. For metabolites, Pearson's and Spearman's correlations, ANOVA, and hierarchical clustering were conducted using cor, aov, and hclust functions in R, respectively (R Core Team, 2012). ANOVA *p*-values were adjusted for false discovery rate (FDR) using the p.adjust function in R (Benjamini and Hochberg, 1995). Heat maps were generated using the corrplot package in R (Wei, 2010). Z scores were calculated using the mean value of a metabolite compared to the mean and standard deviation of the metabolite for the control variety (BTx623). Principal component analysis (PCA) on morpho-physiological and structural traits was performed on data that was mean-centered and scaled to unit variance (UV). PCA on metabolites was performed on data that was mean-centered and Pareto-scaled using SIMCA v14.0 (Umetrics, Umea, Sweden). The O2PLS model was performed in SIMCA and was a regression of morphology and physiology traits (*y* variables, scaled to unit variance) against metabolites (*x* variables, scaled to unit variance).

## Results

### Significant metabolite variation was observed in sorghum leaf extracts among sorghum lines and between “grain” and “biomass” types

Biochemical variation was investigated among the 11 lines of the sorghum panel in order to explore relationships of primary and secondary metabolites to morphological, physiological, and structural carbohydrate traits. Additionally, the sorghum panel was also analyzed by sorghum type, with respect to size/use as (a) grain sorghum (smaller stature) or (b) forage/sweet/biomass sorghum (larger; referred to as “biomass sorghum”).

A non-targeted metabolomics approach was used to evaluate the extent of variation among diverse genotypes without the need to assign compound names to mass spectra. For this study, metabolites were annotated if they correlated to a morpho-physiological trait, described below (for annotation procedures, see Materials and Methods Section “Metabolomics data analysis”). Overall, 6494 and 10,957 molecular features were detected in LC- and GC-MS datasets, respectively. After clustering features, 487 and 694 spectral clusters were generated for LC- and GC-MS datasets, respectively, for a total of 1181 estimated compounds in the sorghum leaf extracts. Of the 1181 compounds, 584 of 694 compounds detected by GC-MS (84.1%), and 372 of 487 (76.3%) detected by LC-MS varied among the 10 sorghum lines (ANOVA, FDR adjusted *p* < 0.05). When grouped by size type, (biomass or grain) 188 of 694 GC-MS-detected and 132 of 487 LC-MS-detected compounds (27.1% for both methods) were significantly different between types (Supplementary Table 1).

**Figure 1:**
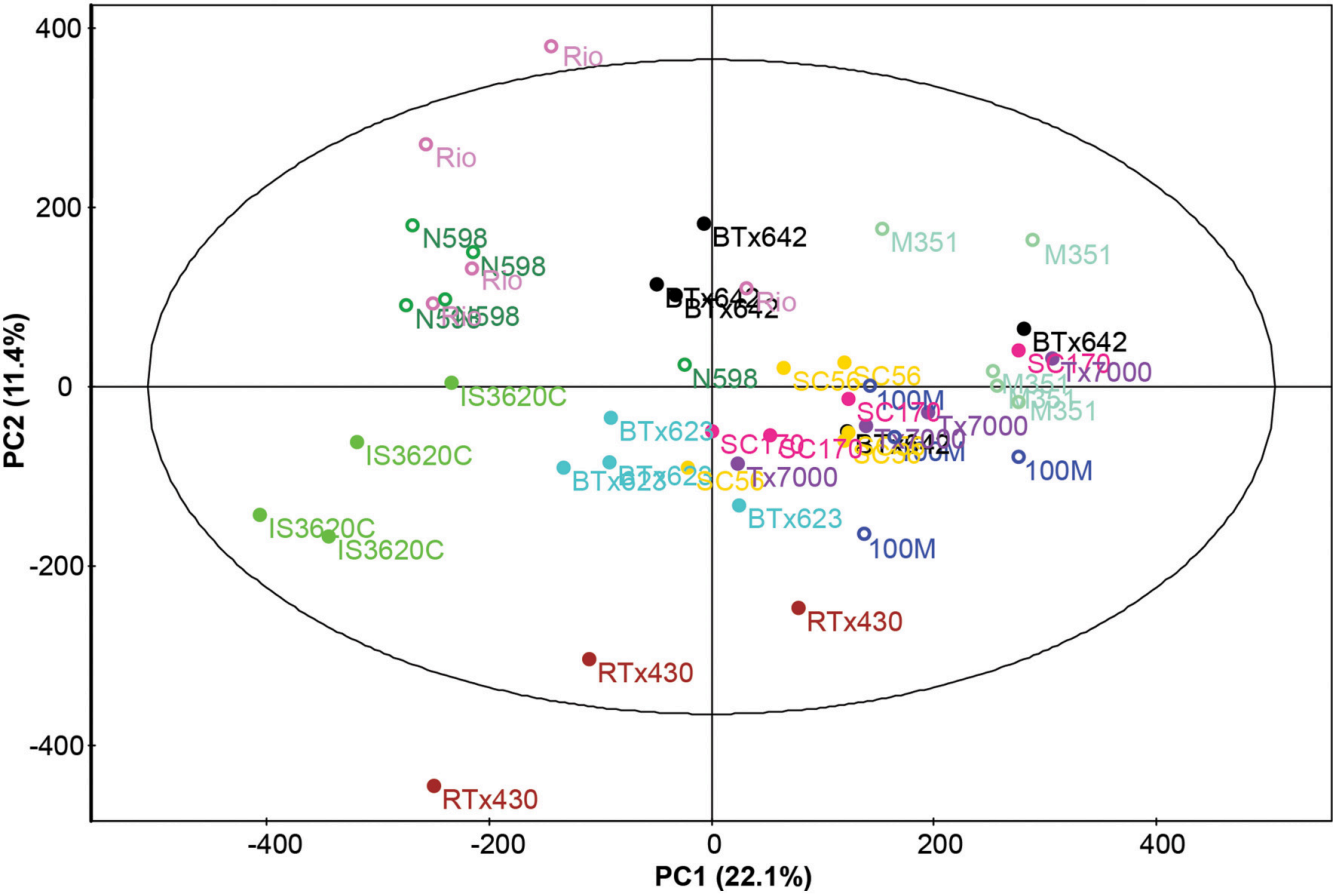
**Scores plot from PCA of the metabolomic analysis of 11 sorghum lines**. Data from GC- and LC- MS analyses were combined. Biomass types are shown with open symbols and grain types with closed symbols.

**Figure 2:**
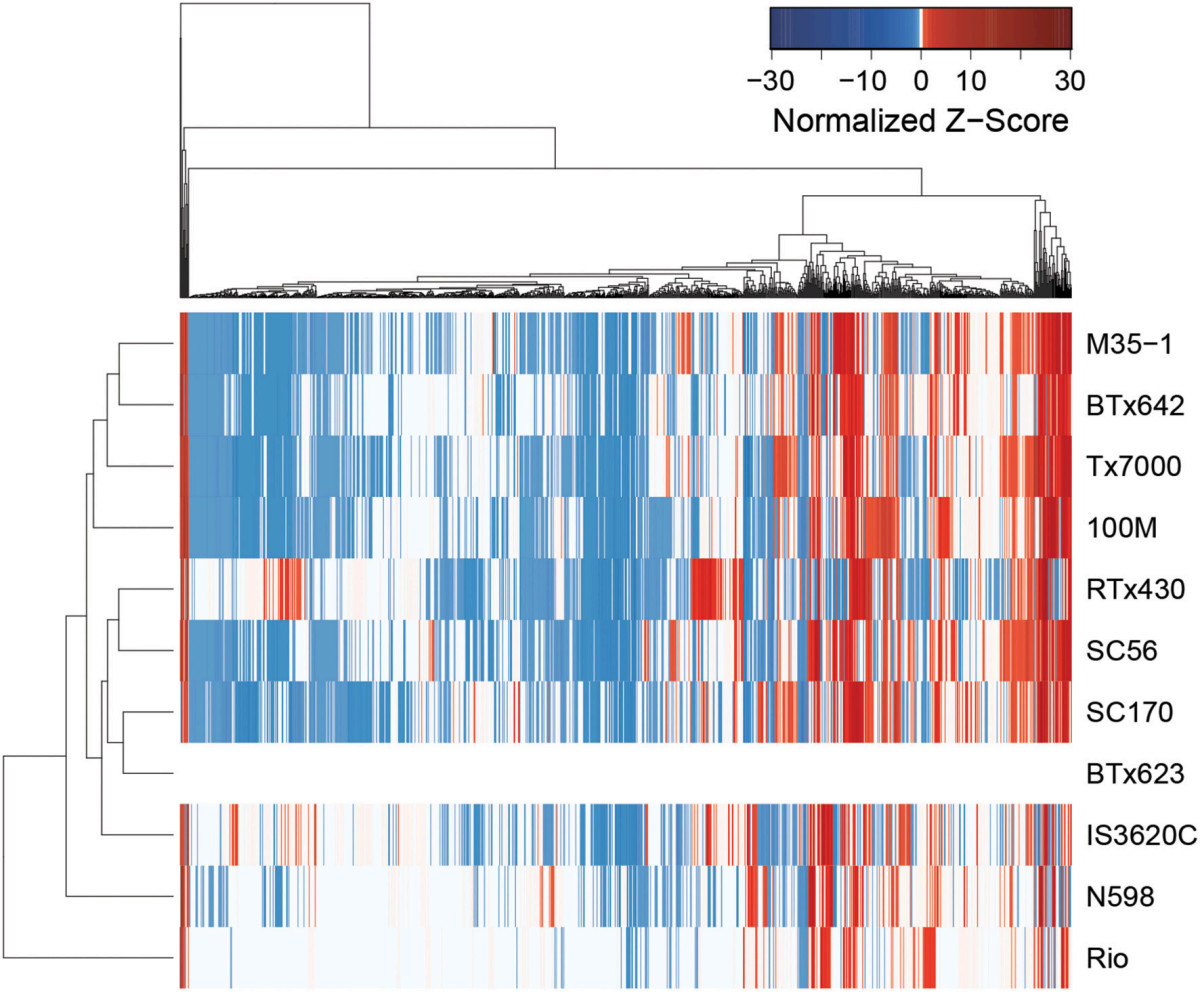
***Z*-scores of metabolite abundance**. The relative abundance of each cluster (metabolite) in each line is normalized to line BTx623 (in which *z*-scores are set to zero and shown in white). Increased abundance relative to control is shown in red and decreased abundance is shown in blue.

*Z*-score and Principal component analysis (PCA) were conducted on these compounds and characterized significant metabolic variation across lines. Nine principal components explained 69.8% of the variation (Table 2). The first two principle components (PCs) are depicted in Figure 1; Although the PCA does not appear to show larger metabolomics trends grain vs. biomass lines, this is likely to be due to a larger influence of “line” on metabolite composition than sorghum “type.” ANOVA was conducted on each PC to determine if metabolites differed among lines and/or sorghum types. All nine PCs varied among sorghum lines (*p* < 0.05) and PCs 2, 5, 6, and 7 varied between sorghum types (“grain” vs. “biomass”; Table 2). As an alternative method to characterize metabolic variation within this sorghum population, all spectral clusters were *z*-transformed using the breeding line BTx623 as the control. The *z*-scores were plotted as a heat map and organized with hierarchical clustering (Figure 2). The heat map provides an overview of the data and indicates metabolic similarity and variation among the breeding lines. Taken together, the metabolomic profiling data indicates significant variation in sorghum leaf metabolite profiles. Multivariate and univariate analyses demonstrate that most metabolites vary by sorghum line and type, and such variation allows for the discovery of associations to morpho-physiological traits.

**Table 2:** **ANOVA on metabolite principal components, by line, and by type**.

**Principal component**	**% Variation explained**	***p*-value by line**	***p*-value by type^a^**
1	22.1	< 0.0001	0.9181
2	11.4	< 0.0001	0.0005
3	7.6	< 0.0001	0.1732
4	6.5	< 0.0001	0.9971
5	5.8	0.0008	0.0039
6	5.1	< 0.0001	0.0044
7	4.2	0.0198	0.0168
8	3.7	< 0.0001	0.6501
9	3.4	< 0.0001	0.5176

a type, “biomass” vs. grain.

### Morphological, physiological, and cell-wall structural traits varied by sorghum line and type

**Figure 3:**
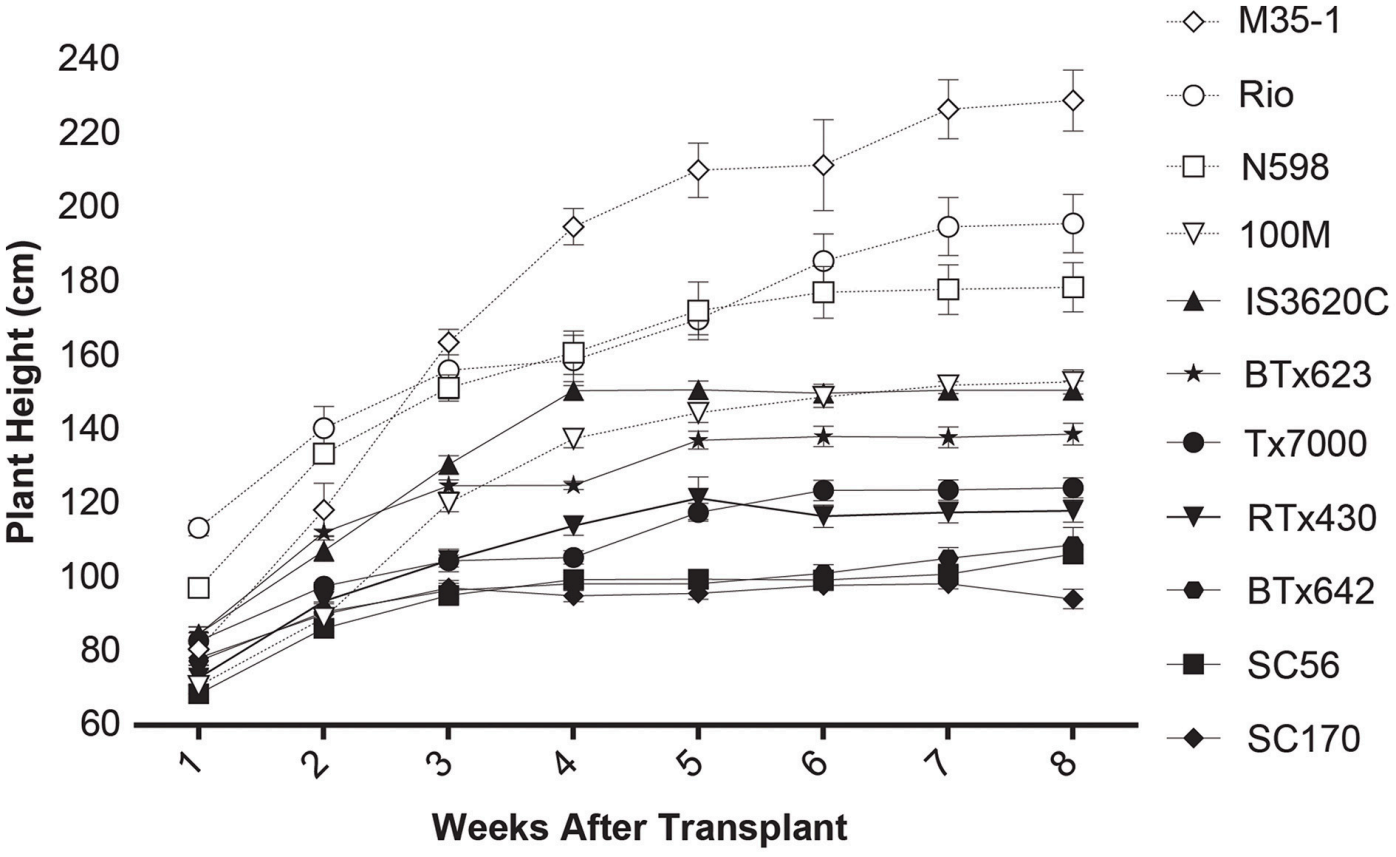
**Growth of 11 sorghum lines over 8 weeks post-transplant, 12 weeks post-germination**. Biomass types are shown with open symbols and dotted lines, grain types with closed symbols and solid lines.

Multiple morphological, physiological, and structural traits were measured in the sorghum panel (Table 1). Significant variation among lines was identified for most traits, although effect sizes varied widely. A summary of the mean morphological and physiological trait values and ANOVA by line is available in Table 3. Variation in growth rate and reproductive maturity impact the duration of sorghum biomass accumulation; partial growth curves for the sorghum panel are presented in Figure 3. The tallest line (*M35-1*) had highest overall growth rates in early, but not later weeks, and also produced most total dry biomass (83.5 g/plant), most of which derived from stems. In contrast, *IS3620C* produced least biomass, though it was mid-range in height; however, it also had smallest stem diameters (Table 3).

**Table 3:** **Physiological and morphological trait means, standard errors, and results of ANOVA by line**.

**Trait**	**100M**	**BTx623**	**BTx642**	**IS3620C**	**M35-1**	**N598**	**Rio**	**RTx430**	**SC170**	**SC56**	**Tx7000**	***F*-Ratio**	**Prob > *F* (*p*)**	**Adj. *R*^2^**
Total dry biomass (g)	71.9± 2.9	66.3± 2.5	63.5± 3.0	58.0± 1.3	83.5± 2.4	72.6± 2.3	80.4± 2.0	58.5± 2.3	63.0± 1.4	59.2± 1.4	65.3± 1.9	15.55	< 0.0001	0.66
Leaf dry biomass (g)	25.2± 3.0	14.8± 0.4	16.2± 0.5	13.9± 0.3	17.4± 0.8	17.3± 0.7	20.0± 0.3	14.7± 0.3	16.2± 0.4	15.2± 0.3	15.5± 0.3	13.76	< 0.0001	0.62
Panicle dry biomass (g)	5.6± 5.6	27.3± 1.3	23.1± 1.9	23.3± 0.8	25.5± 2.2	25.0± 1.1	25.5± 0.7	25.0± 1.4	27.0± 0.4	24.1± 0.7	28.8± 1.2	14.71	< 0.0001	0.65
Stem dry biomass (g)	37.0± 1.1	24.2± 1.0	24.2± 1.0	20.8± 0.5	40.1± 2.7	30.4± 1.3	34.9± 1.9	18.8± 0.8	18.7± 1.2	19.9± 0.7	21.0± 0.6	32.16	< 0.0001	0.80
Plant height (cm)	158.3± 2.0	138.8± 3.0	109.0± 4.7	150.6± 2.5	231.5± 0.2	178.5± 6.6	183.1± 15	118.0± 3.0	94.2± 2.7	106.0± 1.5	124.3± 2.7	37.76	< 0.0001	0.82
Stem diameter (mm)	13.7± 0.28	11.9± 0.40	18.2± 0.54	10.4± 0.25	11.5± 0.41	11.1± 0.28	12.8± 0.28	12.8± 0.43	17.8± 0.47	16.4± 0.53	14.7± 0.48	43.78	< 0.0001	0.84
Avg. individual leaf area (mm^2^)	510.0± 9.9	283.9± 13	330.3± 15	270.4± 11	300.6± 15	268.4± 17	260.4± 16	268.8± 14	286.2± 6.0	322.9± 18	330.0± 11	28.82	< 0.0001	0.78
Leaf harvest index	0.41± 0.042	0.22± 0.005	0.26± 0.012	0.24± 0.006	0.21± 0.007	0.24± 0.005	0.25± 0.006	0.25± 0.006	0.26± 0.003	0.26± 0.005	0.24± 0.005	13.95	< 0.0001	0.63
Panicle harvest index	0.07± 0.070	0.41± 0.007	0.36± 0.013	0.40± 0.005	0.31± 0.029	0.34± 0.010	0.32± 0.011	0.43± 0.006	0.43± 0.008	0.41± 0.007	0.44± 0.007	27.25	< 0.0001	0.78
Stem harvest index	0.52± 0.030	0.36± 0.005	0.38± 0.007	0.36± 0.004	0.48± 0.026	0.42± 0.013	0.43± 0.015	0.32± 0.004	0.31± 0.006	0.34± 0.006	0.32± 0.004	37.72	< 0.0001	0.83
Growth Rt. weeks 1-2 (cm/week)	18.4± 1.4	27.5± 1.6	13.2± 2.3	22.5± 2.1	37.6± 4.2	36.3± 2.5	27.0± 5.0	20.7± 3.1	11.9± 1.7	17.6± 1.9	14.8± 2.2	8.90	< 0.0001	0.50
Growth Rt. weeks 3-4 (cm/week)	24.2± 1.2	6.4± 0.5	3.8± 1.3	21.6± 1.2	38.3± 4.9	13.6± 3.0	8.6± 0.9	10.3± 1.0	2.5± 1.1	6.6± 1.2	3.9± 0.8	25.85	< 0.0001	0.76
ChlA (mg/g)	0.63± 0.05	0.66± 0.07	0.54± 0.05	0.64± 0.04	0.52± 0.03	0.69± 0.02	0.49± 0.03	0.72± 0.08	0.73± 0.06	0.69± 0.03	0.49± 0.04	4.12	0.0006	0.38
ChlB (mg/g)	0.17± 0.02	0.17± 0.02	0.15± 0.02	0.16± 0.01	0.12± 0.01	0.19± 0.01	0.15± 0.01	0.18± 0.02	0.22± 0.02	0.17± 0.01	0.13± 0.02	3.65	0.0015	0.34
ChlTot (mg/g)	0.81± 0.07	0.83± 0.09	0.69± 0.06	0.81± 0.05	0.66± 0.03	0.89± 0.03	0.64± 0.04	0.91± 0.10	0.97± 0.08	0.88± 0.03	0.62± 0.06	4.06	0.0006	0.38
A/B	3.74± 0.18	3.91± 0.12	3.78± 0.30	3.97± 0.16	4.19± 0.08	3.70± 0.12	3.23± 0.08	3.95± 0.28	3.35± 0.14	3.96± 0.10	3.82± 0.26	2.6	0.0154	0.24
A/T	0.78± 0.008	0.79± 0.005	0.78± 0.015	0.79± 0.006	0.80± 0.003	0.78± 0.006	0.75± 0.005	0.79± 0.011	0.76± 0.007	0.79± 0.004	0.78± 0.011	2.51	0.0185	0.23
Ci/Ca	0.319± 0.054	0.273± 0.022	0.377± 0.007	0.360± 0.012	0.362± 0.030	0.240± 0.034	0.288± 0.012	0.268± 0.040	0.312± 0.015	0.455± 0.015	0.309± 0.023	6.30	< 0.0001	0.19
g_s_(mmolH_2_O m^−2^s^−1^)	0.099± 0.014	0.133± 0.010	0.226± 0.004	0.179± 0.006	0.184± 0.008	0.097± 0.007	0.150± 0.010	0.193± 0.009	0.173± 0.011	0.282± 0.013	0.195± 0.009	30.18	< 0.0001	0.57
A(μmolCO_2_m^−2^ s^−1^)	13.13± 0.50	21.39± 1.03	31.83± 0.79	25.93± 0.68	26.07± 0.92	15.87± 0.48	24.06± 1.30	31.71± 0.38	27.70± 2.16	33.91± 1.62	29.883± 1.04	41.04	< 0.0001	0.65
WUE	159.2± 13.4	168.5± 5.79	140.5± 1.30	146.4± 2.84	145.6± 7.11	177.9± 8.58	164.0± 3.27	167.9± 9.39	157.4± 2.93	121.2± 3.43	157.8± 5.67	7.02	< 0.0001	0.22

Ci/Ca ratio of intercellular (leaf) [CO_2_]to ambient [CO_2_]; g_s_, stomatal conductance; A, photosynthesis; WUE, Water Use Efficiency; ChlA, B, Tot, Chlorophylls A, B, and Total, respectively; A/B and A/T, ratio of chlorophyll A to B and A to Total, respectively.

Structural components of plant tissue have important relationships with biomass quality and quantity; therefore NIRS analysis and a calibration model based on wet chemistry were used to examine composition of stem tissue (Table 4). Composition of glucan, xylan, lignin, and ash ranged from 26.8 to 31.3, 16.4 to 19.7, 14.8 to 17.3, and 4.5 to 7.2%, respectively. Total mass/plant of each constituent ranged from 18.9 to 22.5, 9.6 to 15.2, 9.0 to 13.3, and 2.9 to 4.5 g/plant for xylan, glucan, lignin, and ash, respectively.

**Table 4:** **Predicted percentages of biomass constituents, uncertainties, and estimated grams/plant for the stem fraction of each line**.

	**Glucan**			**Xylan**			**Lignin**			**Ash**		
	**(%)^a^**	**UC**	**(g/plant)^b^**	**(%)^a^**	**UC**	**(g/plant)^b^**	**(%)^a^**	**UC**	**(g/plant)^b^**	**(%)^a^**	**UC**	**(g/plant)^b^**
100M	28.3	3.7	20.4	19.7	2.3	14.2	16.3	2.5	11.7	4.5	3.0	3.2
BTx623	30.0	3.3	21.6	16.9	2.1	11.2	16.0	2.2	10.6	5.9	2.7	3.9
BTx642	28.3	3.8	20.4	17.7	2.5	11.2	16.1	2.6	10.2	4.5	3.1	2.9
IS3620C	31.1	3.7	22.4	18.7	2.3	10.9	15.5	2.4	9.0	6.1	3.0	3.5
M35-1	26.8	4.1	19.3	18.2	2.6	15.2	15.9	2.7	13.3	4.6	3.4	3.8
N598	26.3	5.1	18.9	18.0	3.2	13.1	14.8	3.4	10.8	5.8	4.2	4.2
Rio	27.1	3.8	19.5	18.8	2.4	15.1	16.0	2.5	12.9	5.0	3.1	4.0
RTx430	29.2	3.4	21.0	16.4	2.2	9.6	16.7	2.3	9.8	6.4	2.8	3.7
SC170	31.3	2.9	22.5	16.6	1.9	10.5	15.4	2.0	9.7	7.2	2.4	4.5
SC56	30.4	3.7	21.9	19.5	2.3	11.5	17.3	2.4	10.2	4.9	3.0	2.9
Tx7000	29.8	3.9	21.4	17.0	2.5	11.1	15.0	2.6	9.8	6.5	3.2	4.3

UC, Uncertainty of predicted % Glucan, Xylan, Lignin, and Ash composition (see Materials and Methods). Higher UCs reflect greater uncertainty of the predicted percentage.

a Predictions determined via NIRS and mixed feedstock calibration model.

b Estimations based on predicted percentage and actual dry biomass.

**Figure 4:**
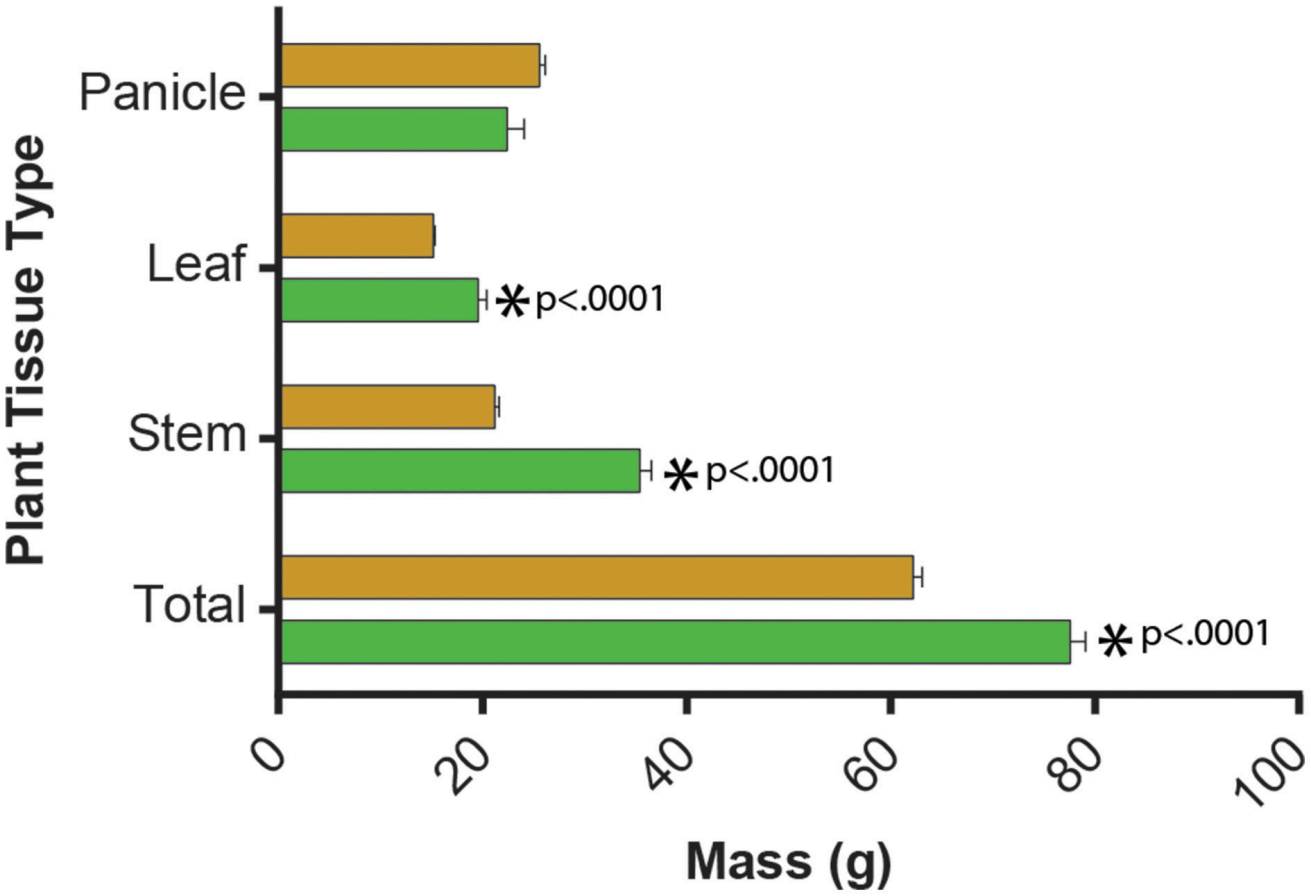
**Histogram of dry biomass according to “biomass” vs. grain types of sorghum**. Grain types are shown in brown, “biomass” types in green. *p*-values are from ANOVA by type as referenced in “Materials and Methods.”

As expected, biomass varied between the two size types (Figure 4), although interestingly, there was no significant difference between panicle biomass of grain and biomass sorghums. However, it should be noted that the line *100M* influenced certain otherwise consistent trait trends between sorghum types; this is discussed at the end of this section. The remaining traits varied significantly between sorghum grain and biomass types, except for the measured or calculated chlorophyll metrics (Table 5). Biomass sorghums were taller and produced more mass; grain sorghums had greater stem diameters, but allocated less overall biomass to stems. Biomass sorghums allocated a large proportion of mass to stems (stem harvest index, SHI) and had highest absolute stem biomass. Grain sorghums had higher leaf harvest indices (LHI), but biomass sorghums produced more overall leaf biomass; Grain sorghum had higher panicle harvest indices (PHI).

**Table 5:** **Physiological and morphological trait means, standard errors, and results of ANOVA by type**.

**Trait**	**Biomass sorghum Mean ± SEM**	**Grain sorghum Mean ± SEM**	***F*-ratio**	***p***	**Adj. *R*^2^**
Plant height (cm)	189.8 ± 6.828	121.6 ± 2.834	106.6	< 0.0001	0.57
Stem diameter (mm)	12.2 ± 0.239	14.6 ± 0.437	13.43	0.0004	0.14
Average Individual leaf area (mm^2^)	323.2 ± 19.02 (276.5 ± 9.52)^*^	300.7 ± 5.997	4.99	0.0287	0.01
Panicle harvest index	0.29 ± 0.022	0.4 ± 0.004	85.13	< 0.0001	0.53
Leaf harvest index	0.26 ± 0.014 (0.23 ± 0.005)^*^	0.2 ± 0.003	4.28	0.0423	0.01
Stem harvest index	0.45 ± 0.012	0.3 ± 0.004	122.5	< 0.0001	0.62
Growth rate, weeks 1–2 (cm/week)	30.6 ± 2.249	18.4 ± 1.070	26.30	< 0.0001	0.24
Growth rate, weeks 3–4 (cm/week)	21.4 ± 2.697	8.0 ± 0.971	29.25	< 0.0001	0.27
Ci/Ca	0.297 ± 0.016	0.341 ± 0.009	6.44	0.0118	0.02
g_s_ (mmol H2O m^−2^s^−1^)	0.134 ± 0.006	0.196 ± 0.006	57.25	< 0.0001	0.20
A (μmol CO2 m^−2^s^−1^)	20.19 ± 0.730	28.36 ± 0.606	78.76	< 0.0001	0.26
WUE	162.9 ± 4.083	150.3 ± 2.319	6.26	0.0131	0.02
ChlA (mg/g)	0.582 ± 0.025	0.632 ± 0.024	1.99	0.1648	–
ChlB (mg/g)	0.159 ± 0.008	0.168 ± 0.007	0.738	0.3942	–
ChlT (mg/g)	0.749 ± 0.033	0.810 ± 0.030	1.7173	0.196	–
A/T	0.777 ± 0.004	0.781 ± 0.004	0.4636	0.4991	–
A/B	3.716 ± 0.096	3.809 ± 0.080	0.5427	0.4647	–

* Starred values calculated with 100M removed as an outlier. Ci/Ca = ratio of intercellular (leaf) [CO_2_]to ambient [CO_2_]; g_s_, stomatal conductance; A, photosynthesis; WUE, Water Use Efficiency; ChlA, B, Tot, Chlorophylls A, B, and Total, respectively; A/B and A/T, ratio of chlorophyll A to B and A to Total, respectively.

**Figure 5:**
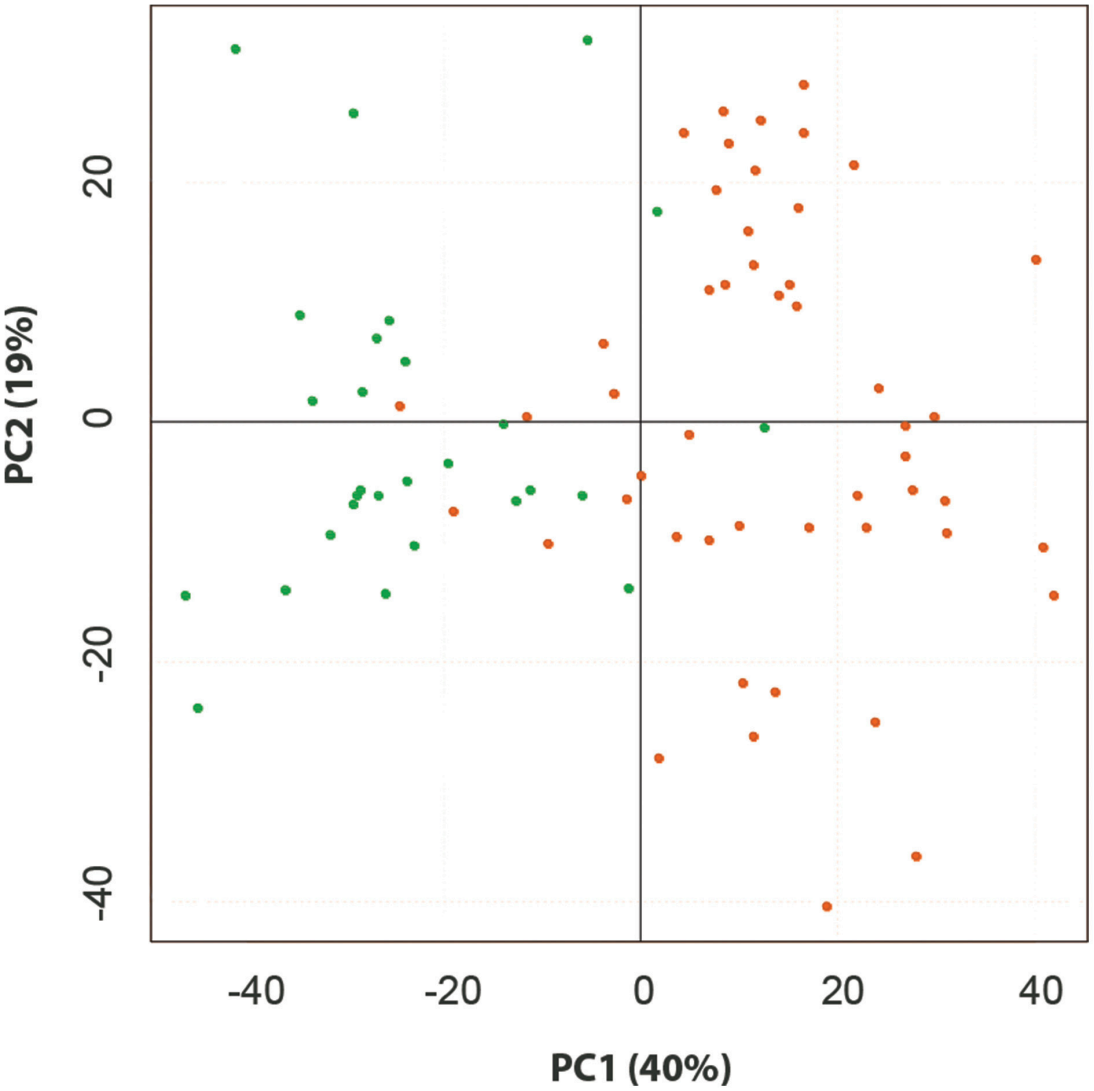
**Scores plot from PCA of the near-infrared spectra of 11 sorghum lines**. Data from NIRS were averaged (*n* = 5–8 per line). Biomass types are shown with green symbols and grain types with brown symbols.

Significant differences in physiological traits were also found between types (Table 5). Smaller grain sorghums had higher rates of photosynthesis, stomatal conductance, and higher Ci:Ca. Biomass sorghums had higher intrinsic water use efficiency (WUE). In addition, PCA was conducted on NIR spectra for stems, and the first two components explained 40 and 19% of variation, respectively. PC1 and PC2 largely separated grain and biomass (Figure 5), indicating cell wall composition profiles to be relatively consistent with sorghum type.

As noted above, line *100M* displayed certain morphological characters which differed from its type (“biomass”). Therefore, for these traits, ANOVA was done both with and without *100M* (Table 5). Without *100M*, biomass lines had smaller leaves than grain lines; inclusion of *100M* skewed the trend significantly in the opposite direction for both leaf area and LHI. Additionally, only one of eight *100M* replicates produced a panicle. Therefore, when analysis included *100M*, panicle weight for biomass sorghum was significantly smaller. However, when *100M* was excluded, no difference existed between absolute panicle weights of biomass and grain-type sorghums, though PHI remained significantly different. Aside from these traits, *100M* was consistent with other biomass types.

**Figure 6:**
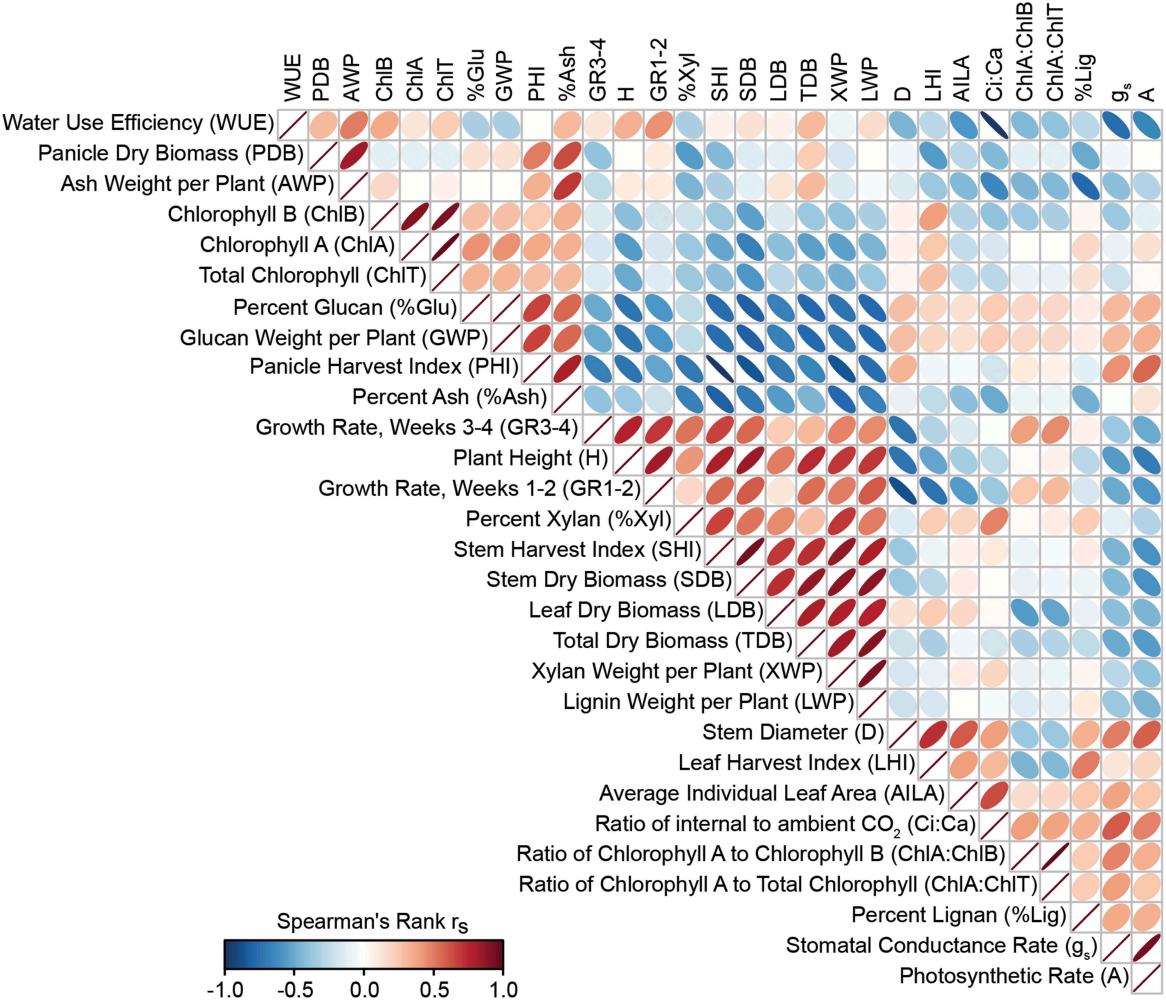
**Heat map of trait to trait correlations**. Heat map of correlations for 29 morphological and physiological traits was created following hierarchical clustering on the spearman *r*_s_-value. Color and ellipse eccentricity denote Spearman's rank correlation *r*_s_ between traits. Correlations with an *r* > |0.602| were significant (*p* < 0.05).

The co-variation of morpho-physiological traits in this panel was investigated using Spearman's rank correlation (Figure 6, Supplementary Table 2). Photosynthesis and stomatal conductance were negatively associated with biomass and other growth traits and physiological processes were correlated with one another. Photosynthesis was positively correlated with stomatal conductance (*r*_s_ = 0.95), Ci/Ca ratios (*r*_s_ = 0.48), and negatively with WUE (*r*_s_ = −0.66). Plant height and growth rate were also negatively correlated with photosynthesis (*r*_s_ = −0.69, −0.49). Chlorophyll A (ChlA) and B (ChlB) were positively correlated with one another (*r*_s_ = 0.90) and ChlA and total chlorophyll (ChlT) were negatively correlated with stem biomass (*r*_s_ = −0.66 and −0.56, respectively). Percent glucan was negatively correlated with total dry biomass (*r*_s_ = −0.78), plant height (*r*_s_ = −0.72), and total stem biomass (*r*_s_ = −0.80) and percent ash was negatively correlated with SHI (*r*_s_ = −0.82).

### Leaf metabolic variation is associated with morpho-physiological phenotypes

**Figure 7:**
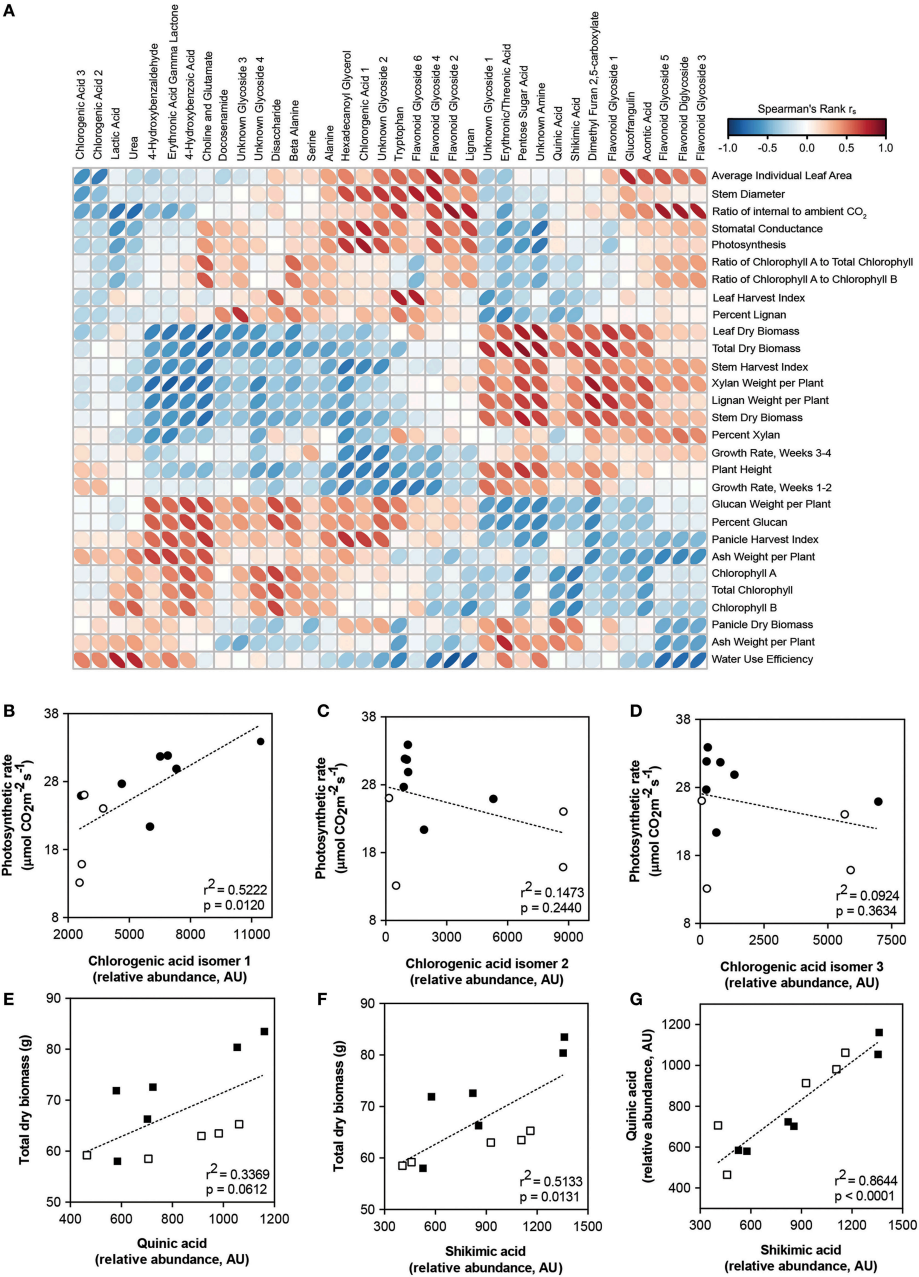
**Correlation of metabolites with morphological and physiological traits. (A)** Heat map with hierarchical clustering of 36 metabolites associated with morphology or physiology in sorghum. Color and ellipse eccentricity denote Spearman's rank correlation *r*_s_ between metabolites and traits. Correlations with an *r* > |0.602| are significant (*p* < 0.05). Numerical values, including *r*_s_ and *p*-values, are given in Additional file 4. **(B–D)** Scatterplots of mean relative abundance for three chlorogenic acid isomers plotted against mean photosynthetic rate for each sorghum line. **(E,F)** Scatterplots of mean abundance for shikimic and quinic acid plotted against mean total biomass for each sorghum line. **(G)** Scatterplot of mean abundance for shikimic acid plotted against mean abundance for quinic acid for each sorghum line. Closed circles, low biomass sorghum lines; open circles, high biomass sorghum lines; closed squares, low photosynthesis sorghum lines; open squares, high photosynthesis sorghum lines. Dashed lines indicate best fit line for linear regression performed on metabolite-trait relationships.

Associations among metabolites and morpho-physiological phenotypes were investigated using univariate and multivariate methods. Spearman's rank correlation was used and characterized 384 of 1181 clusters (32.5%) as associated with at least one morpho-physiological trait (*r*_s_ > |0.5|). Of the 386 metabolites, 36 (~10%) could be annotated by matching mass spectra to several metabolite databases, and are displayed as a heat map following hierarchical clustering with correlations to all measured traits (Figure 7A, Supplementary Table 3). The data indicate sets of metabolites are associated with sets of morpho-physiological traits. The most notable trend was the positive association of glycosylated flavonoids with photosynthesis-related traits (e.g., photosynthesis, stomatal conductance). Organic acids, including erythronic/threonic acid (two compounds with identical mass spectra), lactic acid, and a pentose sugar acid were negatively correlated with photosynthesis. Further, mass spectra that matched to chlorogenic acid (CGA) were detected at three distinct elution times, indicating the presence of three structural isomers. CGA isomer 1 was correlated with both photosynthesis (positive), biomass (negative), and growth rates (negative for both weeks 1–2 and 3–4).

**Figure 8:**
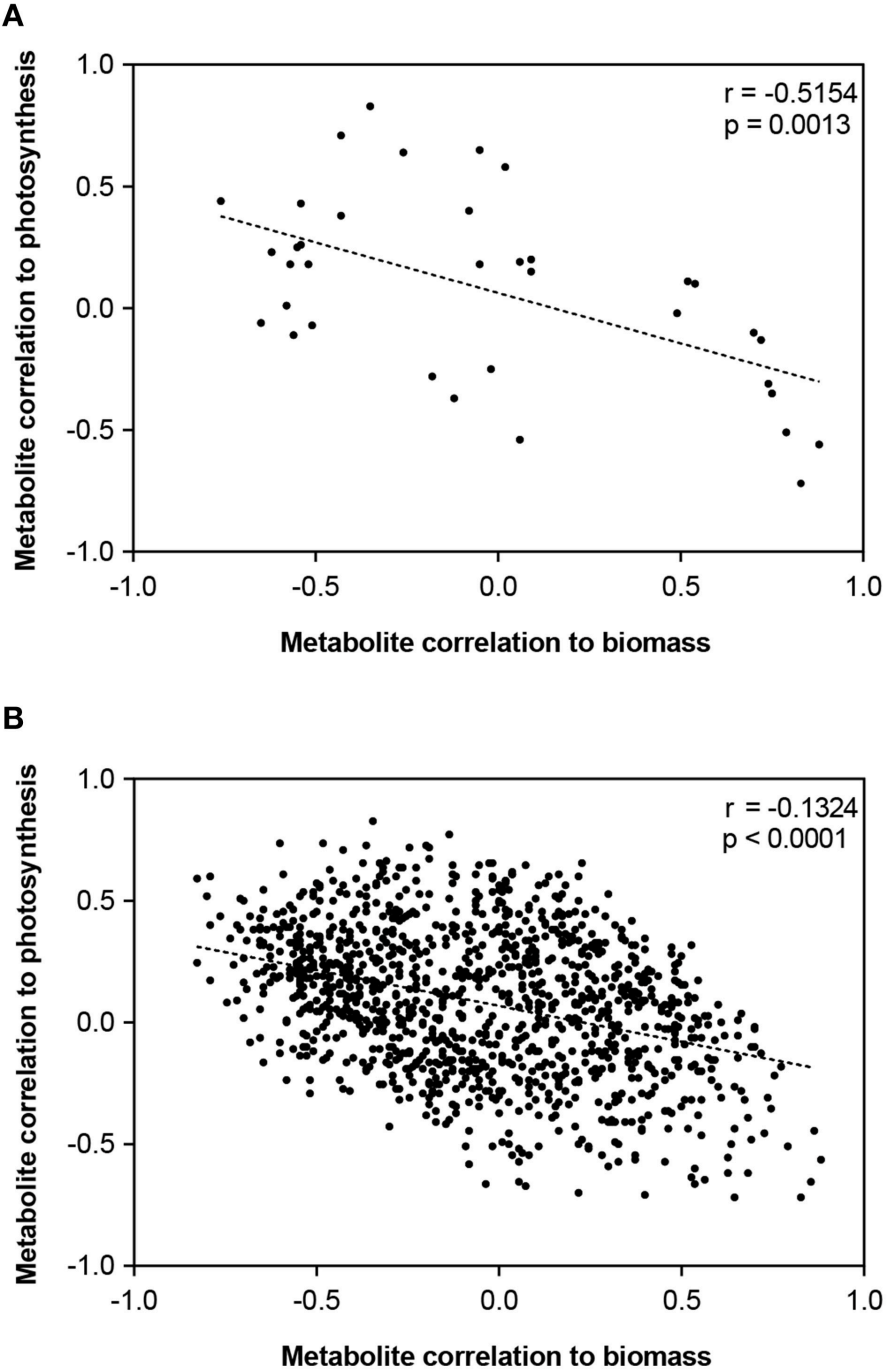
**Association of sorghum leaf metabolome to photosynthesis and biomass**. Spearman's correlation values for the metabolite-photosynthesis relationship plotted against correlation values for the metabolite-biomass relationship. Best fit line for linear regression is used to display the overall trend. **(A)** Scatterplot of correlations for only the 36 annotated metabolites described in Figure 7. **(B)** Scatterplot of correlations for all 1181 detected spectral clusters.

These data were further integrated using O2PLS, a multivariate regression technique based on orthogonal projection to latent structures (OPLS), is a method to integrate two different multivariate datasets (Bylesjo et al., 2007). Here, O2PLS regressed the 20 morpho-physiological traits against 1181 LC- and GC-MS spectral clusters (Trygg, 2002; Figure 8). The O2PLS model resulted in four components that explained 82% of the variation. The O2PLS (Figure 8) indicates associations among morpho-physiological traits, and highlights metabolites that co-vary with these trends. For example, a positive correlation exists between total dry biomass, stem biomass, and HI-stems, and these traits were negatively associated with HI-Panicles. This supports that sorghum biomass is largely driven by carbon allocation toward stems (vs. panicles), and that there are metabolites that follow similar trends.

### Shikimic and chlorogenic acid are potential mediators of carbon assimilation, growth rate, and final dry biomass phenotypes in sorghum

An interesting trend was observed between photosynthesis, growth rates, and final dry biomass. In this study, the lines with the highest photosynthesis (carbon assimilation rates measured at an early time point) had the lowest growth rates in weeks 1–2 and 3–4, as well as the lowest total dry biomass at the end of the experiment. While counterintuitive, several other studies have found negative associations between early photosynthetic measurements and plant growth phenotypes (Wassom et al., 2003; Jahn et al., 2011).

The metabolites that co-varied with these morpho-physiological traits included both primary and secondary metabolites. Of the three CGA isomers, one isomer was negatively correlated to growth rates (1–2 and 3–4 weeks) and total dry biomass (Figure 7B); the other two had slight negative correlations (Figures 7C,D). Shikimic and quinic acid were positively correlated with biomass (Figures 7E,F). In addition, abundances of shikimic and quinic acid within all sorghum lines were highly correlated (Figure 7G). Relative abundances of shikimic, quinic, and chlorogenic acid (isomer 1) among lines are presented in Supplementary Figure 1. Interestingly, metabolites positively correlated to photosynthesis also tended to be negatively correlated to biomass (Figure 8A). When this relationship was explored in the full metabolomics dataset (all 1181 spectral clusters), a significant trend was also observed (Figure 8B).

Taken together, these data indicate shikimate and CGA are associated with the relationship between carbon allocation (photosynthesis) and plant growth (early-developmental growth rates and final dry biomass). This is supported by the associations for these metabolites in both univariate and multivariate statistical models.

## Discussion

Recent research has shown increasing use of both targeted and non-targeted metabolomic approaches to characterize a molecular level of phenotypic variation in both model species such as *Arabidopsis* (e.g., Sulpice et al., 2010) as well in crops such as tomato (Tikunov et al., 2005) and rice (Kusano et al., 2015). In addition to defining a species metabolome, this characterization of variation is also used as a “top-down” approach to better comprehend the complexities of metabolic pathways (Fernie and Klee, 2011). Further, metabolomics are being used to find biomarkers that are indicative of valuable traits in crops such as potato (Steinfath et al., 2010) and barley (Heuberger et al., 2014a) and to examine changes in metabolite profiles as plant immune responses to biotic influence (e.g., the influence of *Fusarium* on soybean root profiles as in Scandiani et al., 2015) as well as in response to abiotic stresses such as drought, as in oats (Sanchez-Martin et al., 2015) or wheat (Bowne et al., 2012). The global profiles of small molecules and subsets therein are also being used in attempts to predict complex traits in the model, *Arabidopsis* (Meyer et al., 2007), although it is becoming increasingly clear that these profiles are likely to be subject to a great deal of environmental influence (Sulpice et al., 2013).

Because of existing genetic tools, the lines examined in this study represent avenues for further discovery and valuable trait exploitation [e.g., sequenced sorghum line *BTx623* (Paterson et al., 2009), re-sequenced lines *Tx7000* and *BTx642* (Evans et al., 2013), or *RTx430*, which is the only commonly transformed sorghum line in this recalcitrant species (Howe et al., 2006; Liu and Godwin, 2012; Wu et al., 2014)].

**Figure 9:**
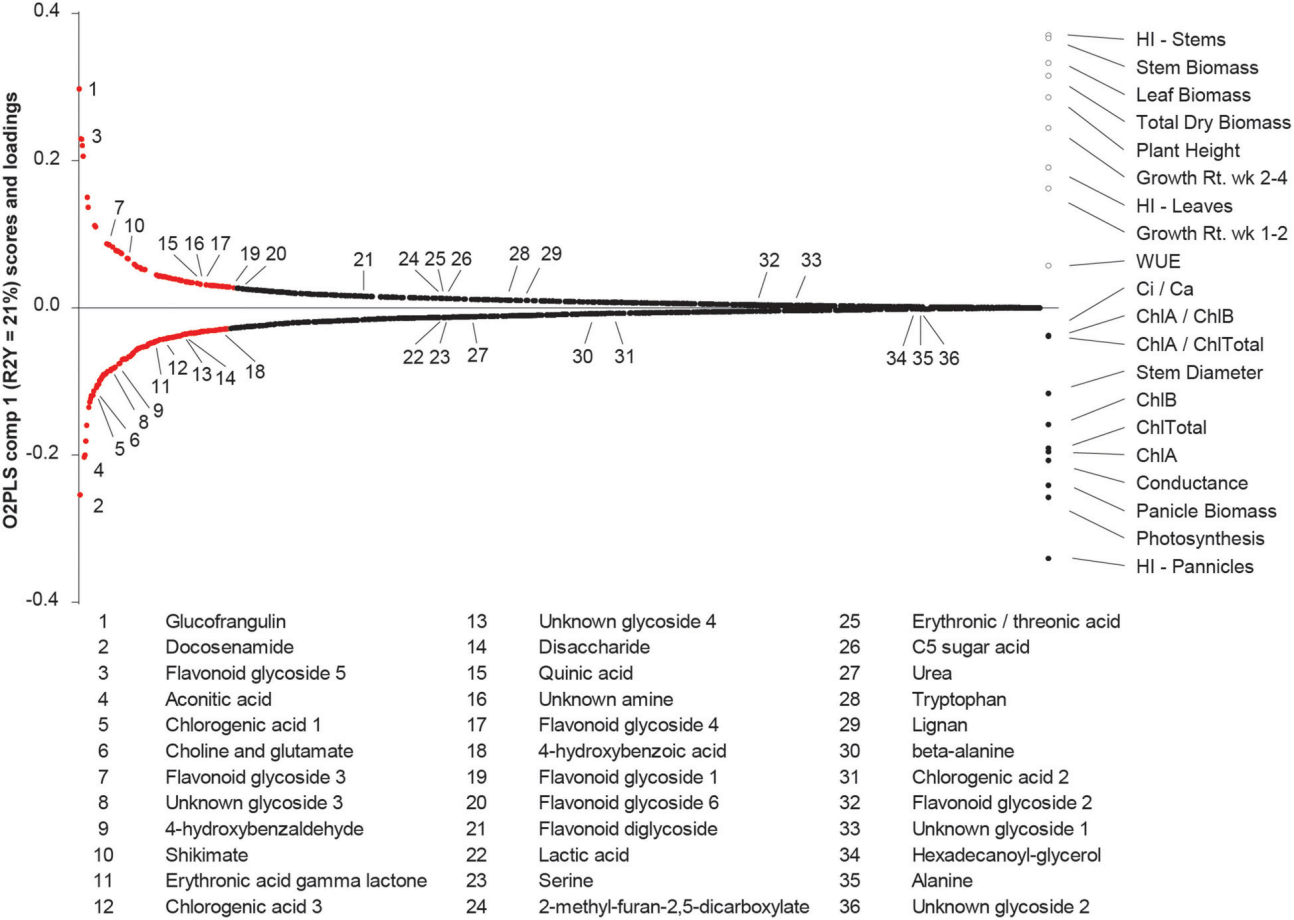
**Prediction of physiological and morphological traits based on metabolic variation**. O2PLS integrated datasets and overlaid trends in metabolites and traits. The *y*-axis represents relative contribution of metabolites to the model (loadings), or relative contribution of traits to the model (scores). Higher or lower loadings or scores denote metabolites and traits with largest contributions to the model and loadings in red were considered to significantly contribute to the model (see *z*-thresholds described in Materials and Methods). The *x*-axis is an ordered list of the 1181 metabolites from highest (left side of *x*-axis) to lowest contributors (right side of *x*-axis). Traits are listed at the right side of the *x*-axis. The positive or negative direction of metabolites and traits indicate co-variation (e.g., stem and leaf biomass co-varies with metabolites 1, 3, 7, 10, etc., and negatively correlate to photosynthesis). The 36 metabolites correlated to at least one trait are denoted.

This study sought to (1) characterize the metabolome variation in this important set of sorghum breeding lines and (2) explore its relationship to morpho-physiological traits related to plant growth and biomass accumulation. Indeed, the data showed that in a phenotypically and genetically diverse species such as sorghum, the metabolome follows suit, exhibiting great variation in small molecule profiles among lines (Figure 3). In addition, correlative relationships among morphological, physiological, and structural traits related to biomass, as well as between metabolite traits and biomass were identified (Figures 6, 7A). It is also of note that certain subsets and whole metabolome patterns demonstrated interesting opposing relationships with biomass, growth rates and photosynthesis (Figures 7B–G, 9) as well as relationships with other physiological and morphological traits (Figures 7A, 8).

The relationship between photosynthetic measurements and subsequent carbon allocation to the accumulation of biomass is notoriously complex. Alteration of photosynthesis is often discussed as a potential mode of crop improvement (Murchie et al., 2009; Zhu et al., 2010), however, relationships between physiological traits and biomass are not necessarily positive (Wassom et al., 2003; Jahn et al., 2011), nor straightforward. It should also be noted there are limitations to measuring photosynthesis in a controlled environment and on a per-leaf area, and these relationships may change at the whole-plant and whole-canopy levels in the field. However, despite this complexity, the results of this study do demonstrate a negative relationship of early growth rates and final biomass to leaf-area based photosynthesis measurements (Figure 6) and are further supported by the relationships of these traits to the leaf metabolome (Figures 7–9). Therefore, our data indicate that the relationship between physiological rates and biomass accumulation may be mediated through both primary and secondary metabolism. Indeed, biomass and photosynthetic rate were among traits with strongest correlations to leaf metabolic profile, highlighting the utility of a metabolomics approach to understand the mechanisms behind biomass regulation.

Specific identified metabolites included Chlorogenic acid (CGA) which was found to be associated with photosynthesis (positive), growth rate (negative), and biomass (negative). CGA is a highly abundant compound and has been demonstrated to have important roles in multiple plant organs including leaves (Sheen, 1973; Mondolot et al., 2006; Clé et al., 2008; Leiss et al., 2009), roots, and root hairs, (Narukawa et al., 2009; Franklin and Dias, 2011), and has roles in diverse processes such as wound response (Ramamurthy et al., 1992; Campos-Vargas and Saltveit, 2002) and cell-wall building (Aerts and Baumann, 1994; Mondolot et al., 2006). In our study, three CGA isomers were identified in leaf extracts. These isomers have been previously described as containing identical MS/MS fragmentation (Xue et al., 2013), but were here separated chromatographically, allowing for independent measurements. One of these three detected isomers had the strongest positive correlation to photosynthesis (Figure 7B); the others were weakly negative. As in this work, other studies have also found isomer-specific metabolite relationships. For example, Xue et al. demonstrated that only one of three chlorogenic acid isomers responded to heat stress and subsequent changes to photosynthesis in *Arabidopsis* (Xue et al., 2013).

CGA is a product of the phenylpropanoid pathway (an intermediate in the lignin pathway), and is a fairly well-characterized scavenger of reactive oxygen species (ROS). In young plant leaves, CGA has been found to localize to chloroplasts (Mondolot et al., 2006); evidence for a protective role against light damage. Further, in green pepper, chlorogenic acid rescued photosynthesis from paraquat-induced inhibition (Laskay and Lakos, 2011). Interestingly, phenolics and spefically CGA, are often inhibitors of plant growth (Einhellig and Kuan, 1971; Li et al., 1993). Thus, these data support that sorhgum lines exhibiting higher rates of carbon assimilation also have high abundances of partiulcar CGA, isomers subsequently inhibiting early growth rates that affect final quantitaties of biomass.

Shikimic acid is another compound involved in important leaf and stem processes, as seen in many studies (e.g., Ossipov et al., 2003; Chaves et al., 2011; Dizengremel et al., 2012; Zhang et al., 2013). Of the metabolites identified in this study, shikimic acid also had one of the strongest positive correlations to biomass. Shikimic acid is a precursor for aromatic amino acids leading into the phenylpropanoid pathway, which is responsible for synthesis of lignin and other secondary metabolites and although it may seem intuitive that more lignin is necessary to support increased biomass, the relationship between them is variable (Hu et al., 1999; Chen and Dixon, 2007; Jahn et al., 2011). Interestingly, shikimic acid has also been observed to be an inhibitor of the Phosphoenolpyruvate Carboxylase (PEPC) enzyme (Colombo et al., 1998) which plays a critical role in the fixation of carbon in C4 organisms. While it remains unclear why a reduction in the amount of photosynthetically fixed carbon would sometimes be associated with an increase in biomass, the competitive inhibition of PEPC may also help to explain why larger sorghums (with more shikimic acid) exhibit lower leaf-level rates of photosynthesis.

Quinic acid, also a constituent of the phenylpropanoid pathway, had a similar correlation profile to shikimic acid (Figure 7G) and is a precursor to CGA. However, in high biomass plants, higher quinic acid levels likely reflect lignin and not chlorogenic acid biosynthesis, as evidenced by lower levels of chlorogenic acid in these lines. A study in *Arabidopsis* found shikimic acid and several other phenylpropanoid metabolites to increase in mutants deficient in lignin biosynthetic enzymes, perhaps as an attempt to recover lignin content (Vanholme et al., 2012). In sorghum, it is therefore likely that increased synthesis of shikimic and quinic acid provide the lignin content necessary to support increased biomass.

This study examined the metabolic variation of an important set of sorghum breeding lines via non-targeted GC- and LC-MS analyses and explored the association of these metabolites and profiles with many measured phenotypes (morphological, physiological, and structural carbohydrate content) relevant to biomass and quality. The sorghum panel exhibited high metabolic variation, some of which co-varied with phenotypic traits; in particular, patterns of and specific metabolites that appear to influence the relationship between carbon assimilation, early stage growth, and final biomass. Because of these notable associations, future research should explore the potential utility of early phenotype prediction via the metabolome to circumvent resource-intense greenhouse and field evaluation of phenotypic traits.

## Author contributions

MT, AH, JK, CC, EW, CB, JP, and CJ wrote the manuscript and had primary responsibility for the final content. CC, CJ, JP conceived the studied and generated the experimental design. MT, CJ, EW collected and analyzed morpho-physiological data. AH, JK, CC, CB collected and analyzed the metabolomics data. MT, AH, JK integrated morpho-physiological and metabolomics data. All authors have read and approved the final manuscript.

## Funding

The work of MT, JK, CC, and CJ was supported by the Colorado State University Agricultural Experiment Station and the Energy Institute at Colorado State University. The work of EW was supported by the U.S. Department of Energy under Contract No. DE-AC36-08GO28308 with the National Renewable Energy Laboratory with funding provided by US DOE Bioenergy Technologies Office (BETO). The content is solely the responsibility of the authors and does not necessarily represent the official views of the Energy Institute at Colorado State University. We would like to acknowledge generous support by the Colorado State University Libraries Open Access Research and Scholarship Fund. The funding bodies did not participate in the design, collection, analysis and interpretation of data; or in preparation of the manuscript.

### Conflict of interest statement

The authors declare that the research was conducted in the absence of any commercial or financial relationships that could be construed as a potential conflict of interest.

## Supplementary material

Supplementary Figure 1 **Differences in relative abundances of key metabolites among lines**. Grain types are shown in brown, “biomass” types in green (line RTx430 had only three replicates, but is a grain line). *p*-values are from ANOVA by line as referenced in “Materials and Methods.”

Supplementary Table 1 ***P*-values from ANOVAs by sorghum type on metabolite clusters**.

Supplementary Table 2 **Spearman's *r*- and *p*-values for trait-trait correlations**.

Supplementary Table 3 **Spearman's *r*- and *p*-values for metabolite-trait correlations**.
