# Supplementary figures and images for: Non-targeted Metabolomics in Diverse Sorghum Breeding Lines Indicates Primary and Secondary Metabolite Profiles Are Associated with Plant Biomass Accumulation and Photosynthesis
Source: Front Plant Sci. 2016 Jul 11;7:953. doi: 10.3389/fpls.2016.00953 (PMC4939745; doi:10.3389/fpls.2016.00953)

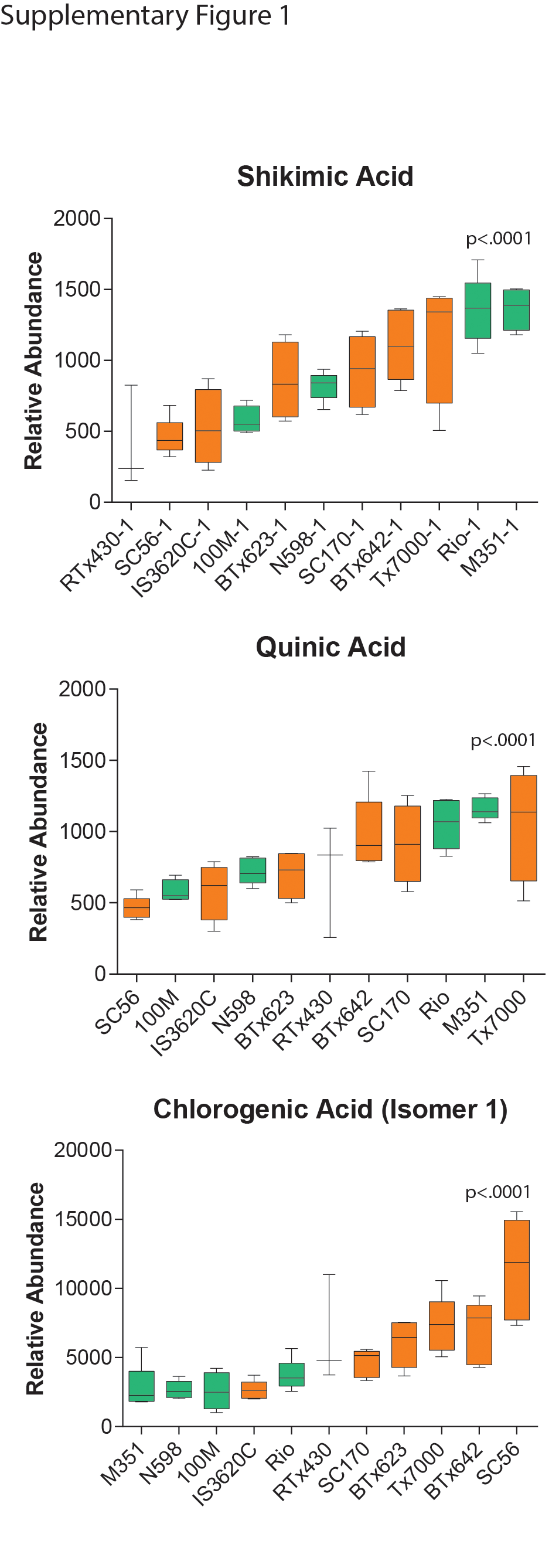

Supplement: Supplementary Figure 1 — Differences in relative abundances of key metabolites among lines. Grain types are shown in brown, “biomass” types in green (line RTx430 had only three replicates, but is a grain line). p-values are from ANOVA by line as referenced in “Materials and Methods.” [file Image1.tif]
